# Self-inflicted Wound of the Throat, Laying Open the Œsophagus—Recovery

**Published:** 1850-08

**Authors:** 


### RECORD OF MEDICAL SCIENCE.

### SURGERY.

*Self-inflicted wound of the throat, laying open the Œsophagus—Recovery.* (Under the care of Mr. Adams.)—Wounds of the throat in persons who attempt to commit suicide, may be of a trifling kind, or cause death instantaneously, either by hæmorrhage or suffocation. There are between these two extremes a great variety of lesions resulting from self-inflicted wounds of the throat, placing the patient in a more or less dangerous situation; among these, the complete division of the thyroid and cricoid cartilages, with a subsequent wound of the œsophagus, are looked upon as extremely hazardous, and the management of such cases requires great care and nicety. It is, however, satisfactory to notice, that an enlightened and close attention to the treatment may triumph over the numerous difficulties which lie in the way of recovery when the wound is of the above mentioned destructive description; and it is our pleasing duty to record a case, lately under the care of Mr. Adams, where favourable results were obtained.

From the notes of Mr. Ball, the house-surgeon, who very courteously afforded us frequent opportunities of seeing the patient, we are enabled to give the following details. On the 28th of February, 1850, a man, about twenty-five years of age, of thin, spare make, and a salesman by trade, was admitted into the hospital with an oblique incision in the anterior part of the throat, extending from above the thyroid cartilage to the fourth ring of the trachea. Both the thyroid and cricoid cartilages, and the three first rings of the trachea, were divided; the knife had passed between the sterno-hyoid and sterno-thyroid muscles, and had slightly lacerated them at their inner edges; the isthmus of the thyroid body was laid bare, but not divided. This desperate wound was inflicted by the patient's own hand while under considerable excitement, with a common table-knife, about an hour previous to admission. The hæmorrhage had been considerable, but had ceased when the patient entered the hospital; the lungs, however, contained a large quantity of blood, which had passed into the trachea, and this fluid, excited by its presence in the lungs, constant cough, with expectoration of the blood through the wound in the throat.

This fact again proves how seldom the suicide succeeds in wounding the common carotid artery or jugular vein, the hæmorrhage mostly proceeding from some of the primary branches of the external carotid. Here it would appear, that the violence used was expended upon the division of the hard bodies above mentioned, viz., the thyroid and cricoid cartilages, the rings of the trachea, and, as will be seen below, part of the œsophagus, these organs being probably rendered prominent by the head having been thrown backwards. The cut was likewise an oblique one, and was therefore less likely to reach to a greater distance posteriorly.

The patient, under these circumstances, was immediately put to bed, his head and shoulders were raised by means of pillows; a silver tube was passed into the trachea to facilitate the ejection of the blood; and lint, wet with cold water, applied to the wound. Mr. Adams saw the patient a few hours after admission, when the breathing was much easier, a large quantity of blood having been expectorated through the tube. Mr. Adams approved of what had been done, and ordered thirty drops of tincture of opium to be given to the patient without a vehicle, so as not to tax the powers of deglutition; but he swallowed this small quantity of fluid with great difficulty, and the attempt excited a violent fit of coughing. The patient made, towards the evening, several unsuccessful attempts to swallow small quantities of milk, but the greater portion of it passed into the trachea and caused violent cough; the milk, at the same time, escaping by the wound in the windpipe.

These phenomena confirmed Mr. Adams in the previous suspicion of wound of the œsophagus; the canula was therefore removed from the wound in the trachea, and an attempt made to pass a flexible tube into the stomach; this was, however, found impracticable, for the tube invariably passed through the wound in the trachea, instead of gliding down from the pharynx into the œsophagus, and excited an alarming amount of irritation. No doubt now remained regarding the wound having reached the œsophagus ; and as no tube could be passed into the stomach, Mr. Adams had recourse to enemata for nourishing the patient. A pint of beef-tea was therefore injected ; the man was allowed to moisten his mouth with a wet rag, and as he breathed quite freely through the wound, it was not thought advisable to replace the canula into it.

This method of administering food by the rectum is invaluable in such cases, and the patient owes his life to this measure ; it is a pity that it is not invariably adopted in analogous circumstances. The case of a child, for instance, was lately mentioned at the Surgical Society of Paris, who had died of inanition. The little patient had had tracheotomy performed upon him to ward off impending suffocation from croup ; as the food subsequently passed through the opening in the trachea, the œsophageal tube was thought of, but could not be used, owing to the inflamed state of the larynx ; the child died. It is not too much to suppose that the child might perhaps have been saved by nourishing enemata.

The difficulty of deglutition in Mr. Adams's patient went on increasing on the second day ; even the swallowing of his saliva gave him great pain ; he was therefore ordered half a pint of beef-tea to be injected into the rectum three times daily. In the night the patient spoke once or twice in a whisper; but strict silence was enjoined, as the effort of speaking excited fits of coughing, which left him greatly exhausted. The bowels having been relieved on the next day after admission, Mr. Adams ordered thirty drops of the tincture of opium to be administered in a starch enema of one ounce, towards the evening, to procure rest. The patient was likewise removed into a private room, as the cold air excited cough. This change to a higher temperature proved very beneficial ; the irritation about the air passages diminished considerably ; there was much less cough ; and a very evident improvement was noticed in the patient's countenance. He, however, was much tormented with thirst, to satisfy which, Mr. Adams ordered an enema composed of a pint of cold milk, and directed a small piece of ice to be placed in the patient's mouth. The wound, in the meantime, went on very favorably, and was dressed solely with lint dipped in cold water.

On the fifth day the patient was able to speak in a whisper without pain or exciting cough, and there was great improvement ; he was nourished entirely by beef-tea and milk enemata, with the administration of thirty drops of tincture of opium by the same means every night, the bowels being kept regular. On the eleventh day after admission, he began to take a small quantity of bread and milk by the mouth, which he succeeded in swallowing without any difficulty. The wound had in the meantime rapidly filled up, and was now about one-fourth of its original size. Beef-tea, milk, rice-pudding, and porter, were soon taken by the mouth, and the patient improved rapidly up to the twenty-third day after the rash act, when symptoms of constitutional disturbance appeared, and pain was complained of both in the head, neck, and shoulders. A purge of calomel and rhubarb, and a blister to the temples, did not succeed in removing these symptoms; the tongue became tremulous and the pulse weak ; the patient was therefore prescribed bark with half a grain of hydrochlorate of morphia at night, and by the assistance of wine, porter, &c., he regained his strength ; the wound healed completely, and the patient was discharged in a very satisfactory condition forty-one days after admission. He was still holding his head rather erect, however, and his voice was somewhat indistinct; but it is to be supposed that with the eventual perfect consolidation of parts within the trachea, and subsequent absorption of exuberant fibrinous deposits, the voice will regain its former tone. It will be noticed that no vessel required tying, and that no secondary hæmorrhage took place; and as certain, not unimportant, branches of the external carotid *must* have been divided, the fact of the cessation of the bleeding will be an additional illustration of the retracting power of arteries, when completely divided. Nor should it be passed unnoticed how well was exemplified in the foregoing case the propriety of avoiding plaster and sutures, as is generally advised by systematic writers when treating of wounds of the thorax.—*Lancet.*

